# RNA sequencing profiles reveal dynamic signaling and glucose metabolic features during bone marrow mesenchymal stem cell senescence

**DOI:** 10.1186/s13578-022-00796-5

**Published:** 2022-05-14

**Authors:** Yanan Sun, Xiao Yu, Xingyu Gao, Chang Zhang, Hui Sun, Kaiyi Xu, Dongxu Wei, Qianwen Wang, Haiying Zhang, Yingai Shi, Lisha Li, Xu He

**Affiliations:** 1 The Key Laboratory of Pathobiology, Ministry of Education, College of Basic Medical Sciences, Jilin University, Changchun, 130021 Jilin China; 2 The High School Attached to Northeast Normal University, Changchun, 130021 Jilin China

**Keywords:** RNA sequence, Glycolysis, MSCs, Senescence, Metabolism

## Abstract

**Background:**

Stem cell senescence is considered as a significant driver of organismal aging. As individuals age, the number of stem cells is declined, and the ability to proliferate and survive is also weakened. It has been reported that metabolism plays an important role in stem cell self-renewal, multilineage differentiation, senescence and fate determination, which has aroused widespread concerns. However, whether metabolism-related genes or signalling pathways are involved in physiological aging remain largely undetermined.

**Results:**

In the current study, we showed 868 up-regulated and 2006 down-regulated differentially expressed genes (DEGs) in bone marrow mesenchymal stem cells (MSCs) from old rats in comparison with that from young rats by performing RNA sequence. And DEGs functions and pathways were further selected by function enrichment analysis. The results indicated that the high expression of DEGs might participate in cell differentiation, growth factor binding and etc., while the down-regulated DEGs were majorly enriched in metabolism process, such as the cellular metabolic process and mitochondria. Then, we screened and verified DEGs related to glucose metabolism and investigated the glycolysis levels. We identified that glucose uptake, lactate secretion, ATP production and relative extracellular acidification rates (ECAR) were all diminished in MSCs from old rats. More importantly, we conducted microRNA prediction on the key DEGs of glycolysis to elucidate the potential molecular mechanisms of glucose metabolism affecting MSC senescence.

**Conclusions:**

Our study unravelled the profiles of DEGs in age-associated MSC senescence and their functions and pathways. We also clarified DEGs related to glucose metabolism and down-regulated glycolysis level in age-associated MSC senescence. This study will uncover the metabolic effects on regulating stem cell senescence, and provide novel therapeutic targets for ameliorating age-associated phenotypes.

**Supplementary Information:**

The online version contains supplementary material available at 10.1186/s13578-022-00796-5.

## Background

In the light of the report about ageing and health from the World Health Organization (WHO), between 2015 and 2050, the proportion of the world's population over 60 years will nearly double from 12 to 22%. Notably, ageing is closely related to the increasing population. Ageing is a natural, inevitable and complex physiological process characterized by progressive structural damage, loss of function, declined coordination and physiological integrity of tissues, which ultimately gives rise to an elevated risk of death [1]. Unfortunately, as age increases, the incidence of various age-related diseases is also elevated, such as diabetes, cardiovascular and cerebrovascular diseases, and malignancies as well. Therefore, how to improve “Healthy Ageing” merits urgent exploration.

Stem cell senescence is the relatively new theory of molecular mechanisms underlying ageing, which may be a risky factor for the reduced number and function of stem cells. As individuals age, the microenvironmental changes may be a driving force to promote stem cell senescence. Simultaneously, the number of stem cells decreases, and the ability to proliferate and survive is also weakened [2]. MSCs have the property of self-renewal and commit to multiple cell lineages, such as bone, cartilage, adipose, muscle, tendon, stroma and neuronal cells, which make them overwhelmingly useful tools in tissue engineering and regenerative medicine [3]. However, MSCs at early passage from the elderly exhibited the senescence-like characteristics, the multilineage differentiation potentials and proliferation capacity also attenuated [4]. This may cause some organ function loss, and restrict the efficacy of autologous stem cell transplantation in elderly patients. Hence, maintaining MSC functions in an adverse aged bone marrow microenvironment awaits specific clarification.

As one of the basic features of organisms, energy metabolism is a reactive steady-state system that can meet the cellular energy needs in specific phases [5]. Recently, it has been reported that cellular metabolism plays an important role in stem cell self-renewal, multilineage differentiation, senescence, and regulation of stem cell fate, which has caused widespread concerns [6–8]. Besides, mitochondrial respiratory function analysis, metabolomics and proteomics have revealed that adult stem cells such as MSCs [9, 10], hematopoietic stem cells (HSCs) [11–13] and neural stem cells [14] mainly rely on glycolysis to produce ATP, which exists in all cellular organisms and is critical for life. It also has been well-established that the glycolytic levels of HSCs from old rats [15] and senescent myoblasts [16] were declined, while that of germline stem cells was improved [17]. However, whether the glucose metabolism of senescent MSCs is mainly dependent on glycolysis, how MSC senescence is modulated by glycolysis, and which genes or signalling pathways play the leading roles in this process, none of the above is largely clear.

In our previous study, we demonstrated that intracellular nicotinamide adenine dinucleotide (NAD^+^) content significantly lowers in MSCs obtained from old rats than those acquired from young rats [4]. Consistently, elevated NAD^+^ synthesis is critical determinants to delay MSC replicative senescence [18]. Decreased osteoblast differentiation potential is associated with attenuated NAD^+^-Sirt1 signalling in MSCs obtained from 8-week-old male C57BL/6 mice [19]. As an important metabolic coenzyme, NAD^+^ plays an indispensable position in the catabolism of energy substrates in most cells. The redox cycle of NAD^+^ provides hydrogen ions for numerous metabolic pathways, such as glycolysis and oxidative phosphorylation. Multiple steps in the glucose metabolism pathways involve the conversion of NAD^+^ to NADH and regeneration of NAD^+^. In addition, we have found the cellular metabolic process of senescent MSCs has changed significantly by the targeted mRNA enrichment analysis of age-related circular RNAs (circRNAs) in our latest article [20]. As mentioned above, MSCs mainly rely on glycolysis to produce ATP. Therefore, we hypothesized that the metabolic pathways may govern a regulatory role during MSC senescence.

In the current study, we showed differentially expressed genes (DEGs) in MSCs from old rats in comparison with MSCs from young rats by performing RNA sequencing, and DEGs functions and pathways were further selected by function enrichment analysis. Then, DEGs related to glucose metabolism were screened and verified, and the glycolytic levels were investigated by glucose uptake, lactate secretion, ATP production and relative extracellular acidification rates (ECAR). Moreover, we conducted microRNA prediction on the differentially expressed key genes of glucose metabolism to elucidate the potential molecular mechanisms of glucose metabolism involved in MSC senescence.

## Results

### Characterization and validation of MSCs obtained from old rats

**Fig. 1:**
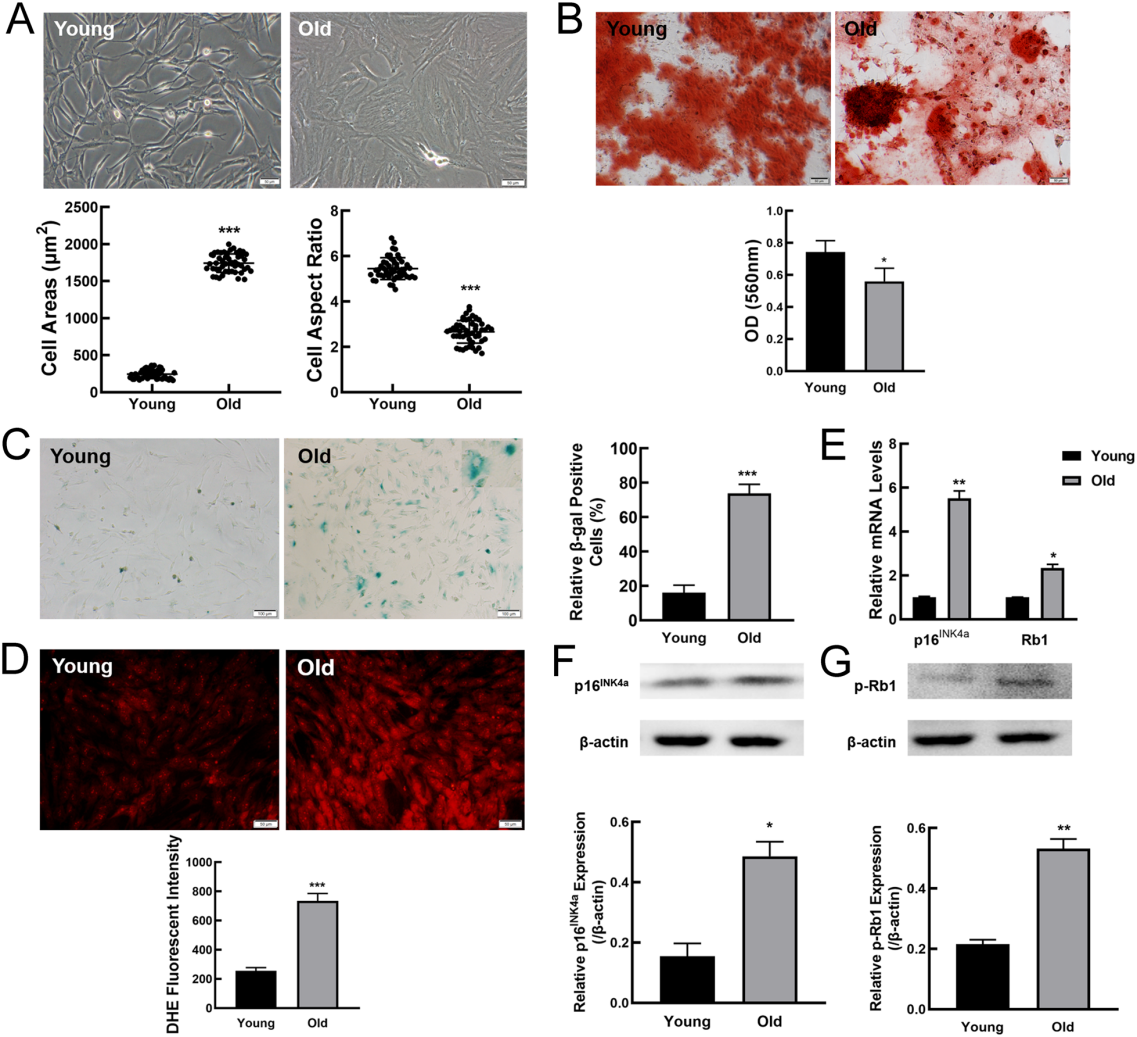
Accelerated ageing phenotypes in MSCs from old rats. **A** Representative bright-field images showing the morphology of MSCs from young and old rats and analysis of cell aspect ratio and cell surface area. Scale bar, 50 µm. **B** Determination of osteogenic differentiation in MSCs from young and old rats by Alizarin Red S staining (scale bar = 50 μm). **C** SA-β-gal staining and the percentages of SA-β-gal-positive cells. Scale bar, 100 μm. **D** Intracellular ROS levels and statistical analysis of ROS fluorescent intensity. Scale bar, 50 μm. **E** The expression of senescence marker genes at mRNA levels by RT-qPCR. **F**, **G** Western blot was performed to examine p16 ^INK4a^ and phospho-Rb1 protein expression levels. β-actin was used as a loading control. All data were presented as mean ± SEM (error bars), n = 3, ***P < 0.001, **P < 0.01, * *P* < 0.05

In previous study, we successfully obtained MSCs by whole bone marrow adherent method, and the morphological characteristics of the primary MSCs from young and old rats were similar [4]. To determine if MSCs from young rats after three passages in vitro presented replicative senescence, MSCs at passage 1 (P1) and passage 3 (P3) were assessed by morphology and senescence-associated β-galactosidase (SA-β-gal) staining. As shown in Additional file 4: Fig. S1A, P1 and P3MSCs displayed similar morphological characteristics, with long and fusiform shape and flocked arrangement. SA-β-gal staining revealed that a small number of blue-stained MSCs at P1 and P3 were observed (Additional file 4: Fig. S1B). These results demonstrated that P1 and P3 MSCs did not undergo significant senescence. Then, we investigated whether P3MSCs become senescent along with individual age. Compared to P3MSCs from young rats, P3MSCs from old rats showed senescent morphological features, such as irregular shapes, enlarged and flattened cell bodies, unclear boundary and noticeable particles in the cytoplasm (Fig. 1A). Statistical analysis of morphology indicated the cell surface area of MSCs from old rats extremely increased, while the aspect ratio was reduced (Fig. 1A). Cell surface antigens commonly used to identify MSCs were further analyzed by flow cytometry. We found that both MSCs from young and old rats are positive for mesenchymal progenitor markers including CD44, CD90, and CD105, and negative for CD31 and CD45 (Additional file 4: Fig. S1C). Multilineage differentiation is one of the most important hallmarks of stem cells, and our data showed that MSCs from old rats presented attenuated osteogenesis compared with young controls (Fig. 1B).

To confirm whether cellular senescence occurred with increasing age, SA-β-gal staining was performed. Representative cell images showed that MSCs from old rats exhibited enhanced expression of SA-β-gal (Fig. 1C). Statistical analysis indicated that the ratio of SA-β-gal positive cells in MSCs from old rats was significantly elevated when compared to that in MSCs from young rats (Fig. 1C). To further clarify that MSCs from old rats were senescent cells, the other methods were used to validate. As shown in Fig. 1D, there was a much weaker reactive oxygen species (ROS) fluorescent intensity in MSCs from young rats. And statistical analysis by Image J software obtained similar results. In addition, senescence markers including p16^INK4a^ and Rb1 expression at mRNA (Fig. 1E) and protein levels (Fig. 1F and G) were greatly up-regulated in MSCs from old rats as compared to young rats. Together, these results indicated MSCs gradually become senescent with the increase of donor age.

### Gene expression alterations of MSCs obtained from old rats

**Fig. 2:**
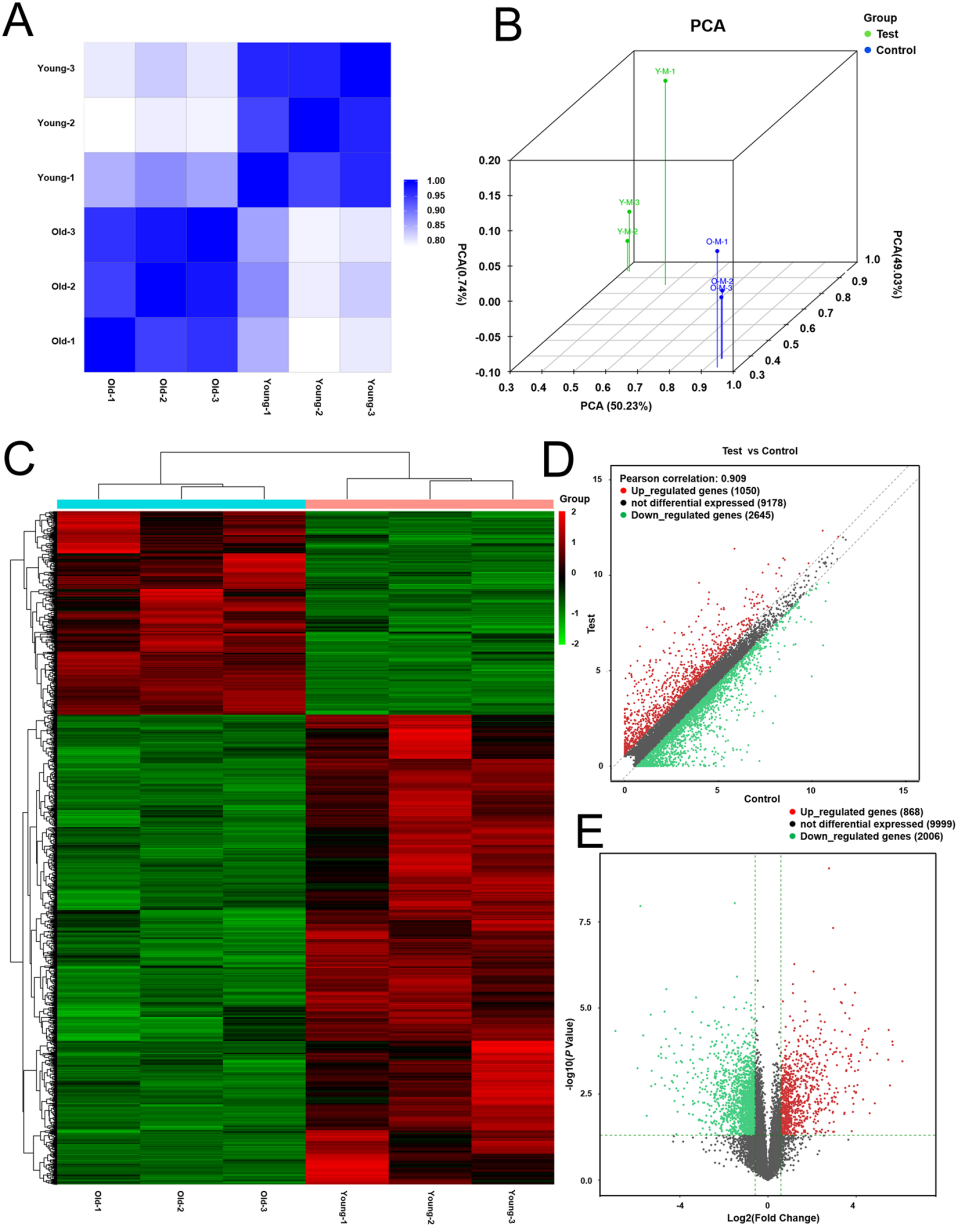
Gene expression analysis of MSCs from young and old rats. **A** Pearson correlation heatmap of mRNAs in MSCs from young and old rats (n = 3). **B** Principal component analysis (PCA) of the gene changes. **C**, **D** The heatmap and scatter plots showing the differential expression profiles of genes between the two study groups and the homogeneity in each group. **E** Volcano plot of DEGs. The green and red dots in the plot represent the DEGs with statistical significance. The red color represents the up-regulated gene, the green color represents the down-regulated gene, and the black represents the gene with no significant change

To investigate whether there are gene transcription changes between young and senescent MSCs, genome-wide RNA sequencing (RNA-seq) was performed. MSCs from young rats as controls, MSCs in old group were regarded as test group for analysis. In order to exclude inter-individual heterogeneity with small sequencing samples, we performed principal component analysis (PCA) and correlation analysis based on sequencing data. The correlation analysis showed significant gene alterations between MSCs from old and young rats (Fig. 2A) and little difference among samples within the same group (Fig. 2B), which suggests that biological variations in gene expression data is strongly associated with age-effect. Differentially expressed genes were identified with a cut‐off of average FPKM ≥ 0.5 and fold change > 1.5. The results showed that 1050 up-regulated and 2645 down-regulated genes between MSCs from young and old rats (Fig. 2C). On this basis, we defined genes satisfying fold change > 1.5 and *P* value < 0.05 as DEGs. The analysis of sequencing data allowed identifying 868 up-regulated and 2006 down-regulated DEGs in MSCs from old rats in comparison with MSCs from young rats (Fig. 2D and E). The detailed information including number and fold change of the DEGs was shown in Table 1. The percentage of significant up-regulated genes (fold change > 3) accounted for 31.22% (including fold change > 3 accounted for 14.86% and fold change > 5 accounted for 16.36%) of total up-regulated genes, which was more than twice that of significant down-regulated genes (fold change < 0.3). In general, our data showed that there are more down-regulated genes than up-regulated genes in old group, while the percentage of significant down-regulated genes is lower in comparison with significant up-regulated genes.

**Table 1:** Numbers and percentage of up/down-regulated DEGs

	Up-regulated			Down-regulated		
	Fold change	Fold change	Fold change	Fold change	Fold change	Fold change
	> 5	> 3	> 1.5	< 0.2	< 0.3	< 0.67
Numbers of DEGs	129	142	597	146	148	1712
Percentage (%)	14.86	16.36	68.78	7.28	7.38	85.34
Total	868			2006		

Among the genes affiliated with stem cell senescence, the top 10 up/down-regulated genes were listed in Table 2. The majority of top 10 up-regulated genes had a directly or indirectly negative influence on cell cycle and cell growth, such as cartilage intermediate layer protein (CILP), four and a half LIM domains 1 (FHL1), transforming growth factor, beta 3 (TGFB3) and myosin heavy chain 11 (MYH11). The down-regulated genes were associated with metabolism and inflammation including aldo–keto reductase family 1, member C14 (AKR1C14), solute carrier family 1 member 3 (SLC1A3), retinol binding protein 4 (RBP4) and metallothionein 2A (MT2A), C–C motif chemokine ligand 20 (CCL20), interleukin 1 receptor antagonist (IL1RN), respectively. This is consistent with the characteristics of stem cell senescence and the expression of age-related factors.

**Table 2:** Details of the top 10 up/down-regulated genes

Gene Name	Chromosome	Fold change	*P* value	FDR
Up-regulated				
CILP	8	68.414	3.530E−04	0.017
FHL1	X	50.450	1.184E−04	0.013
TGFB3	6	49.868	9.427E−05	0.012
ACTA2	1	46.407	1.783E−03	0.029
ITGA8	17	44.424	4.355E−05	0.010
MYH11	10	43.851	3.185E−04	0.017
MYOCD	10	30.975	2.123E−04	0.016
ELN	12	28.741	5.912E−03	0.046
FAM150A	5	24.827	3.258E−04	0.017
IL1RL1	9	24.463	1.143E−02	0.062
Down-regulated				
SFRP1	16	0.008	4.656E−05	0.010
AKR1C14	17	0.017	5.622E−04	0.020
CDH2	18	0.018	1.088E−08	5.212E−05
MT2A	19	0.019	6.261E−05	0.011
CSCL6	14	0.022	1.357E−02	0.067
SLC1A3	2	0.025	1.558E−05	0.006
RBP4	1	0.025	1.860E−03	0.029
LUM	7	0.026	6.696E−04	0.021
CCL20	9	0.029	1.638E−03	0.028
IL1RN	3	0.032	2.500E−04	0.016

*FDR* the adjusted *P* value, *P* *value* the *P* value of the F-statistic for gene from young and old MSCs, *ACTA2* actin alpha 2, *AKR1C14* aldo–keto reductase family 1 member C14, *ALKAL1* ALK and LTK ligand 1, *CCL20* C–C motif chemokine ligand 20, *CDH2* cadherin 2, *CILP* cartilage intermediate layer protein, *CXCL6* C-X-C motif chemokine ligand 6, *ELN* elastin, *FHL1* four and a half LIM domains 1, *IL1RL1* interleukin 1 receptor-like 1, *IL1RN* interleukin 1 receptor antagonist, *ITGA8* integrin subunit alpha 8, *LUM* lumican, RBP4 retinol binding protein 4, *MT2A* metallothionein 2A, *MYH11* myosin heavy chain 11, *MYOCD* myocardin, *SFRP1* secreted frizzled-related protein 1, *SLC1A3* solute carrier family 1 member 3, *TGFB3* transforming growth factor beta 3

### Gene Ontology (GO) analysis

**Fig. 3:**
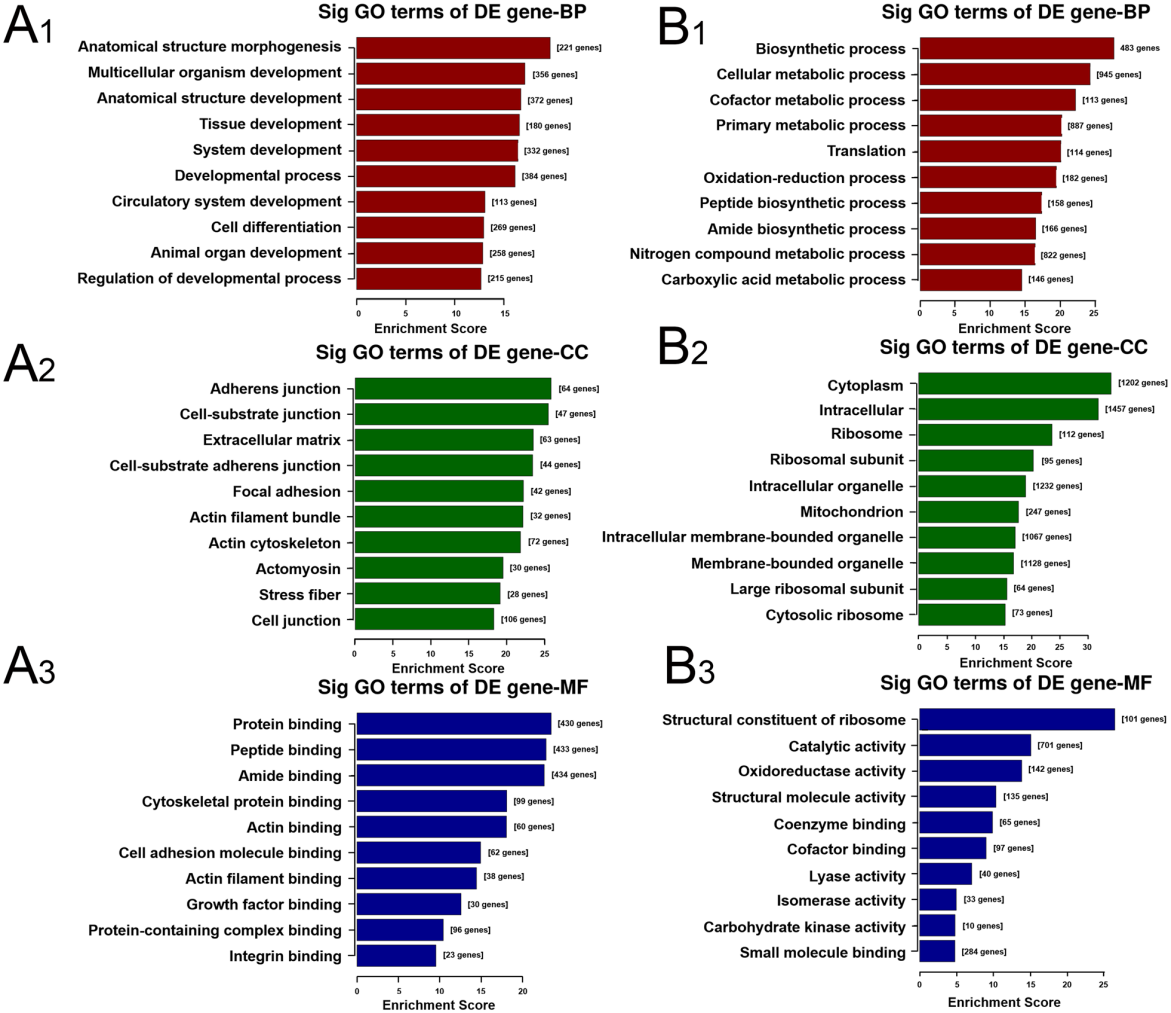
GO analysis of the DEGs between MSCs from young and old rats. **A** GO analysis showing the top 10 ranked up-regulated terms enriched in MSCs from old rats. A1, A2 and A3 represent biological process, cellular component and molecular function, respectively. **B** GO analysis (biological process, cellular component and molecular function) of significantly down-regulated genes in MSCs from old rats

In order to elucidate the functional and biological characteristics of DEGs, we performed GO analysis including biological process, cellular component and molecular function. The top 10 significant terms (ranked by *P* value) to preliminarily cluster the function of DEGs were selected, as shown in Fig. 3 and Additional file 1. In the biological process, anatomical structure morphogenesis, multicellular organism development, anatomical structure development, tissue development and system development were overrepresented (Fig. 3A1), while the metabolism process such as cellular metabolic process, cofactor metabolic process, primary metabolic process, nitrogen compound metabolic process and carboxylic acid metabolic process were remarkably down-regulated (Fig. 3B1). In cellular component, terms related to cellular matrix or adhesion such as adherens junction, cell-substrate junction, extracellular matrix, cell-substrate adherens junction and focal adhesion were elevated (Fig. 3A2), and down-regulated genes were obviously associated with the component of cellular organelle including ribosome, ribosomal subunit, intracellular organelle, mitochondrion and intracellular membrane-bounded organelle (Fig. 3B2). In addition, molecular function terms such as protein binding, peptide binding, amide binding, cytoskeletal protein binding and actin binding were enhanced in MSCs from old rats (Fig. 3A3), while low-expressed genes were mainly enriched in structural constituent of ribosome, catalytic activity, oxidoreductase activity, structural molecule activity and coenzyme binding (Fig. 3B3). These changes in gene expression underlie young and senescent MSCs, further supporting the roles of cellular matrix and metabolism in regulating stem cell senescence.

### KEGG pathway enrichment analysis

**Fig. 4:**
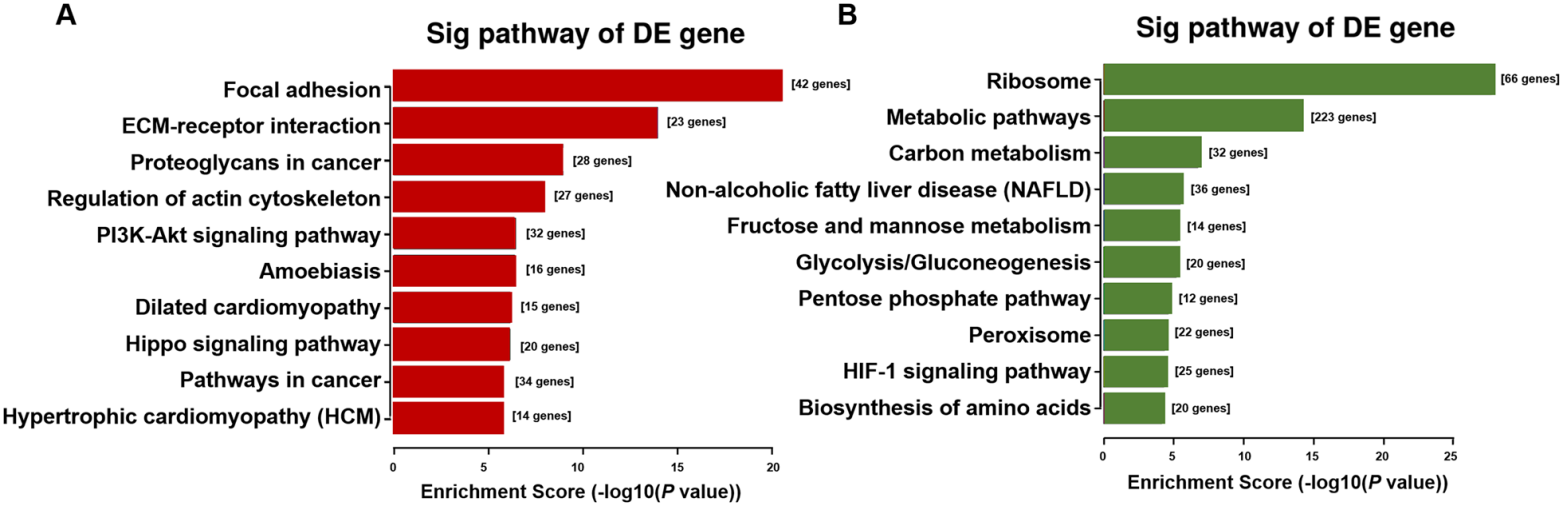
KEGG pathway enrichment analysis of DEGs in old rats compare to that in young rats. **A**, **B** The most significantly up- and down-regulated pathways between young and senescent MSCs. The red color indicates the up-regulated pathways in old rats, while the green color represents the down-regulated pathways

To comprehensively understand the age-related pathways, we performed KEGG pathway enrichment analysis, and the results were shown in Fig. 4 and Additional file 2. We discovered that focal adhesion, ECM-receptor interaction, proteoglycans in cancer, regulation of actin cytoskeleton, PI3K-Akt signaling pathway were up-regulated (Fig. 4A). However, the levels of metabolism in old groups showed a significant reduction compared to those in young groups, which could be proved by the down-regulated DEGs enrichment in metabolic pathways, carbon metabolism, non-alcoholic fatty liver disease (NAFLD), fructose and mannose metabolism, glycolysis/gluconeogenesis and pentose phosphate pathway (PPP) (Fig. 4B). These findings revealed that the metabolic levels of MSCs gradually decrease as aging occurs.

### Declined glycolysis levels in MSCs obtained from old rats

**Fig. 5:**
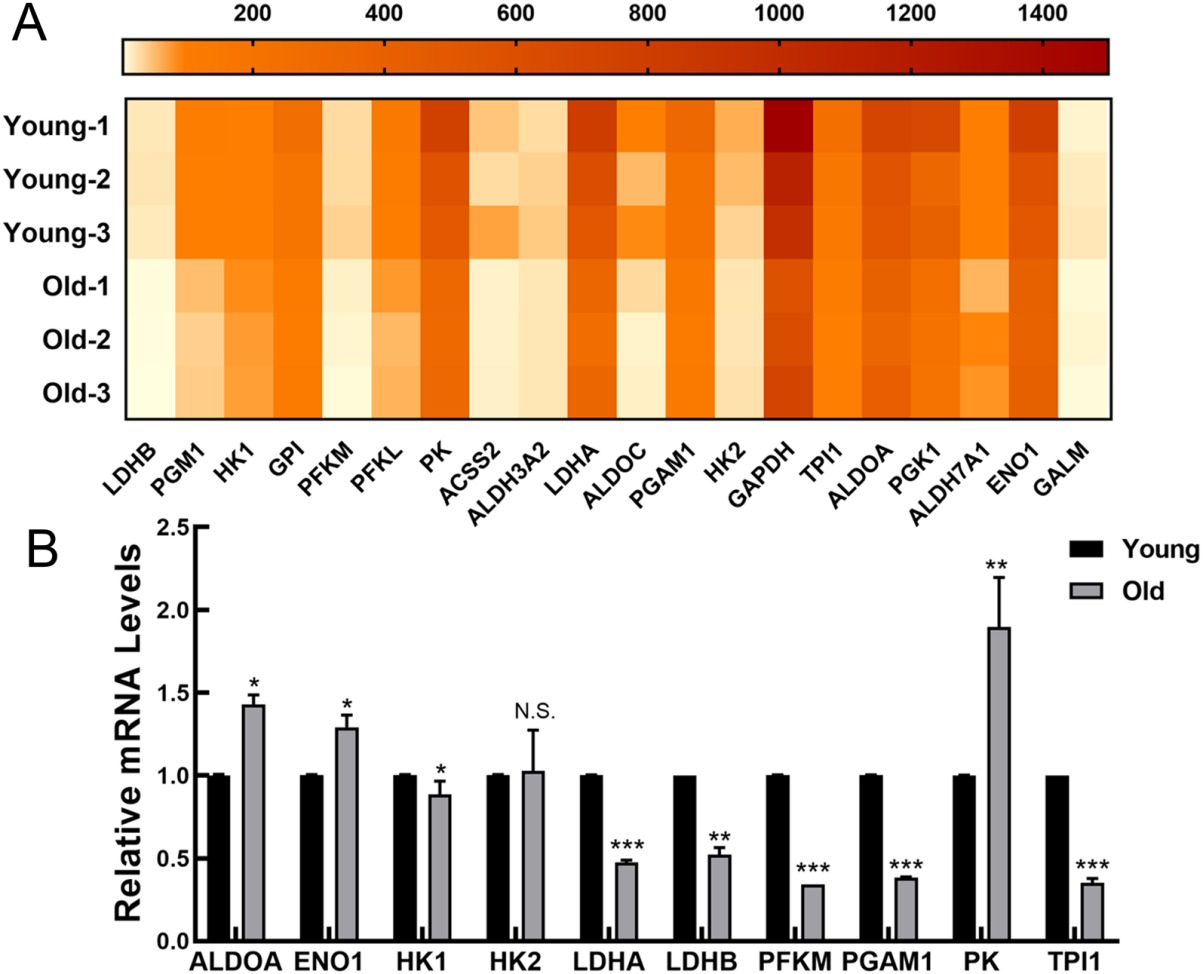
The expression levels of glycolysis related genes were reduced in MSCs from old rats. **A** The heatmap showing the average PFKM in MSCs from young and old rats by RNA sequence. **B** RT-qPCR results for relative mRNA expression of glycolysis related genes in MSCs. All data were presented as mean ± SEM (error bars), n = 3, ***P < 0.001, **P < 0.01, *P < 0.05, N.S. indicated no statistical value

To further verify the alteration in glycolytic levels of MSCs from old rats, we selected the genes from the glycolysis/gluconeogenesis pathway, which ranked 6th in the KEGG pathway enrichment analysis. The gene selection criteria include relatively high expression level; a cut‐off of absolute *P* value < 0.5 between two groups; KEGG pathway; or published literatures indicate that this gene might be associated with glycolysis processes. The details of glycolysis/gluconeogenesis pathway related genes were list in Table 3. Among these genes, lactate dehydrogenase B (LDHB), hexokinase 1 (HK1), glucose-6-phosphate isomerase (GPI), phosphofructokinase in muscle (PFKM), phosphofructokinase in liver (PFKL), pyruvate kinase (PK), lactate dehydrogenase A (LDHA), aldolase-fructose-bisphosphate C (ALDOC), phosphoglycerate mutase 1 (PGAM1), hexokinase 2 (HK2), glyceraldehyde-3- phosphate dehydrogenase (GAPDH), glyceraldehyde-3-phosphate dehydrogenase (TPI), aldolase-fructose-bisphosphate (ALDOA) and enolase 1 (ENO1) were coding catalytic enzymes of the glycolytic pathway. Surprisingly, 4 of the top 10 genes in this pathway were the key rate-limiting enzymes in glycolysis, including HK1, PFKM, PFKL and PK. In addition, LDHB and LDHA were essential for the conversion of pyruvate to lactic acid, therefore they were the guarantee of glucose metabolism. The RNA-seq results demonstrated that the relative expression levels of these genes from old rats were significantly lower than those from young rats (Fig. 5A), which indicated the glycolytic levels decreased as multicellular organisms aged. To confirm the gene expression patterns obtained from RNA-seq, real time quantitative reverse transcription polymerase chain reaction (RT-qPCR) was performed by applying sequencing samples. As shown in Fig. 5B, the expression of glycolytic enzymes including HK1, PFKM, LDHA, LDHB, TPI and PGAM1 at mRNA levels displayed a remarkable reduction, while the expression levels of ENO1, ALDOA and PK mRNA were increased. Contrary to the sequencing results, there is no obvious change in HK2 expression. The data indicated that glycolysis may exert the regulatory effects on MSC senescence and the roles of genes whose sequencing results consistent with RT-qPCR results in senescent MSCs merit urgent exploration.

**Table 3:** The details of glycolysis/gluconeogenesis pathway related genes

NO.	Gene name	Chromosome	Fold change	*P* value	FDR	Regulation
1	LDHB	4	0.324	9.015E-05	0.012	Down
2	PGM1	5	0.376	0.002	0.029	Down
3	HK1	20	0.638	0.002	0.030	Down
4	GPI	1	0.483	0.003	0.036	Down
5	PFKM	7	0.378	0.003	0.036	Down
6	PFKL	20	0.368	0.005	0.043	Down
7	PK	8	0.529	0.008	0.052	Down
8	ACSS2	3	0.338	0.011	0.060	Down
9	ALDH3A2	10	0.634	0.011	0.060	Down
10	LDHA	1	0.493	0.012	0.062	Down
11	ALDOC	10	0.249	0.012	0.064	Down
12	PGAM1	1	0.496	0.014	0.069	Down
13	HK2	4	0.508	0.015	0.070	Down
14	GAPDH	4	0.546	0.016	0.072	Down
15	TPI1	4	0.435	0.019	0.079	Down
16	ALDOA	1	0.664	0.022	0.084	Down
17	PGK1	X	0.471	0.027	0.095	Down
18	ALDH7A1	18	0.608	0.029	0.100	Down
19	ENO1	5	0.657	0.031	0.102	Down
20	GALM	6	0.579	0.044	0.124	Down

*FDR* the adjusted *P* value, *P* *value* the *P* value of the F-statistic for gene from young and old MSCs, *AcCSS2* acyl-CoA synthetase short-chain family member 2, *ALDH3A2* aldehyde dehydrogenase 3 family member A2, *ALDH7A1* aldehyde dehydrogenase 7 family member A1, *ALDOA* aldolase fructose-bisphosphate, *ALDOC* aldolase fructose-bisphosphate C, *ENO1* enolase 1, *GALM* galactose mutarotase, *GAPDH* glyceraldehyde-3-phosphate dehydrogenase, *PFKL* phosphofructokinase liver type, *PFKM* phosphofructokinase muscle, *PGK1* phosphoglycerate kinase 1, *PGM1* phosphoglucomutase 1, *PK* pyruvate kinase, *GPI* glucose-6-phosphate isomerase, *HK1* hexokinase 1, *HK2* hexokinase 2, *LDHA* lactate dehydrogenase A, *LDHB* lactate dehydrogenase B, *PGAM1* phosphoglycerate mutase 1, *TPI1* triosephosphate isomerase 1

**Fig. 6:**
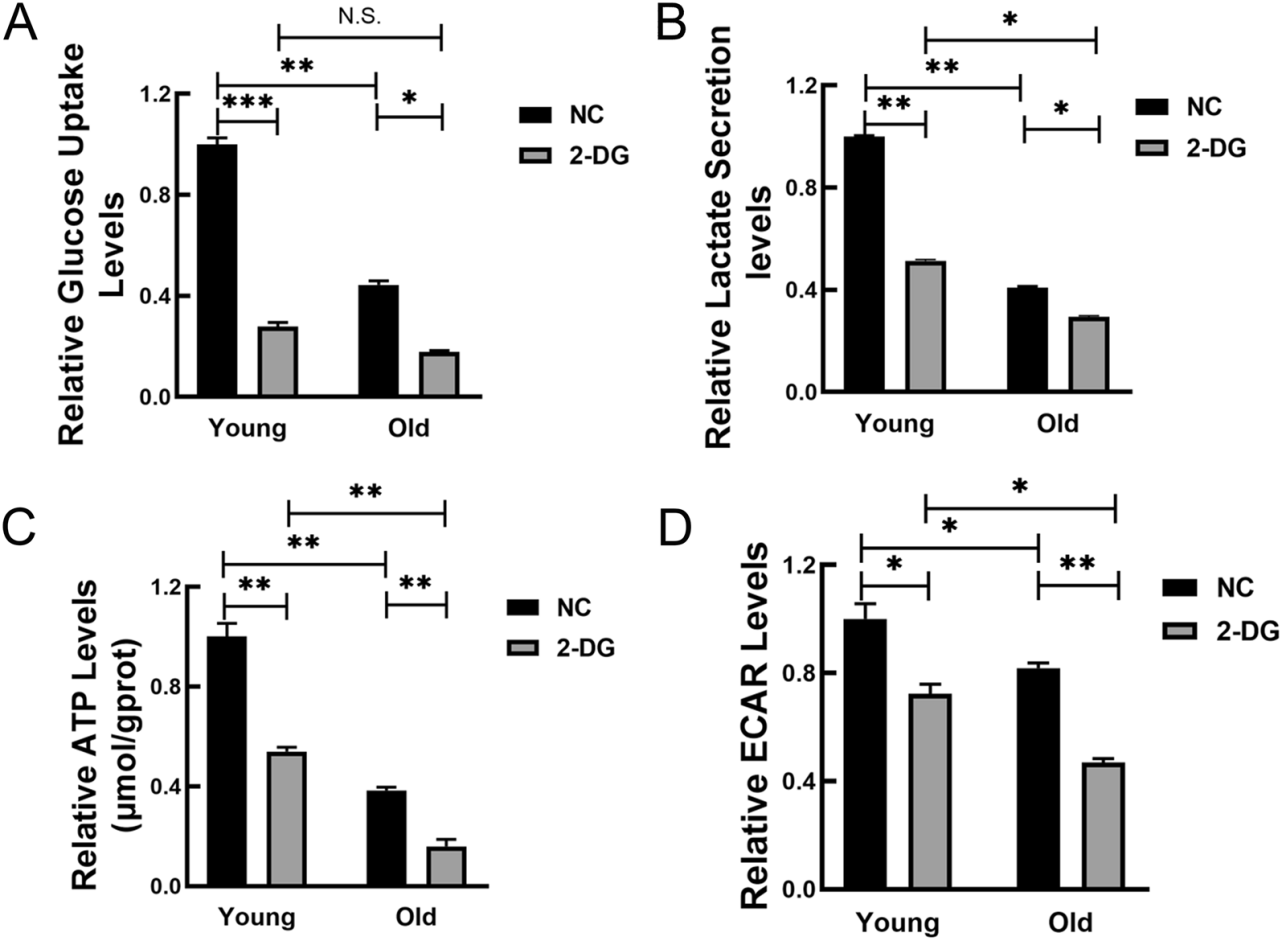
The levels of glycolysis were decreased in MSCs from old rats. **A**–**D** Relative glucose uptake (**A**), lactate secretion (**B**), relative ATP levels (**C**) and ECAR levels (**D**) were measured in MSCs from young and old rats. All data were presented as mean ± SEM (error bars), n = 3, ***P < 0.001, **P < 0.01, *P < 0.05, N.S. indicated no statistical value

Then we try to figure out whether the glycolysis levels decreased during MSC senescence. For this purpose, glucose uptake and lactate levels were examined in both young and senescent MSCs. As shown in Fig. 6A and B, MSCs from old rats displayed a lower uptake of glucose and lactate secretion compared to those in young cells. Consistently, ATP production and the extracellular acidification rate (ECAR) were also substantially reduced in MSCs from old rats (Fig. 6C and D). These data suggested that the glycolysis levels decreased in MSCs obtained from old rats. 2-Deoxy-D-glucose (2-DG), a glucose structural analog, effectively inhibits the glycolysis by inhibiting hexokinase. After 2-DG treatment, the glucose uptake, lactate secretion, ATP production and relative ECAR were all blunted in MSCs from young and old rats (Fig. 6). And the glycolysis levels in MSCs from old rats may be altered more obviously compared with those from young rats. The above results demonstrated that glycolysis may be the main glucose metabolism in bone marrow-derived MSCs from rats.

### MiRNA prediction of age-related metabolic genes

**Fig. 7:**
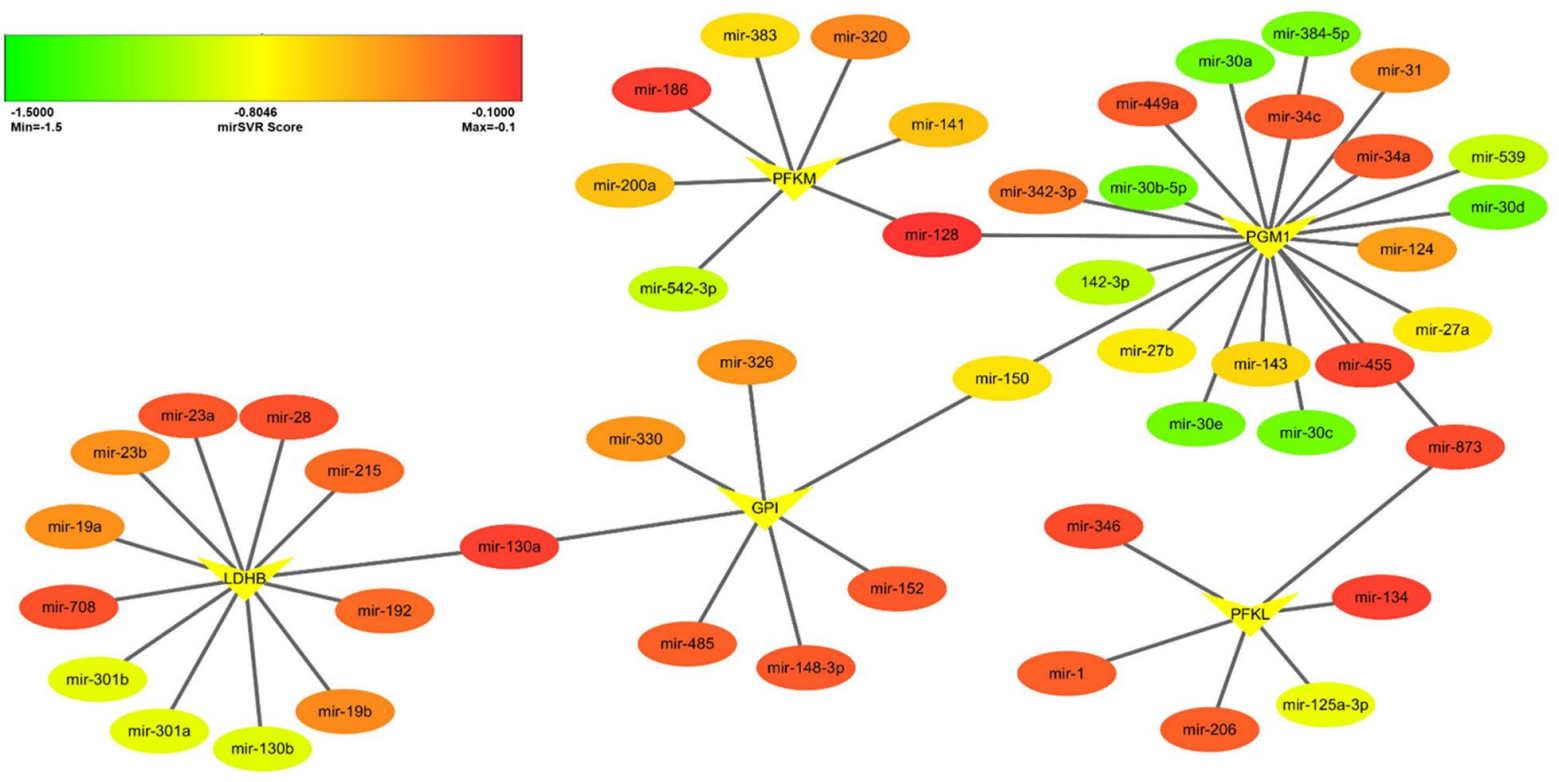
The potential miRNAs of age-related metabolic genes accessed by Cytoscape. Target miRNAs were selected according to PhastCons ≥ 0 and mirSVR ≤ -0.1 by online database (microRNA.org). In this figure, ovals represent miRNAs and darts represent mRNAs

MicroRNAs (miRNAs) play a vital role in the regulation of a variety of biological processes, including cell senescence, proliferation and differentiation, and energy metabolism [21]. To explore glycolysis-related miRNAs, we performed target miRNA predictions on the top 5 glycolysis-related genes including LDHB, PGM1, GPI, PFKM and PFKL. Target miRNAs were selected according to PhastCons ≥ 0 and mirSVR ≤ − 0.1. The mRNA-miRNA interaction network was constructed using Cytoscape software version 3.7.1. (Fig. 7). The established network comprised 5 mRNAs and 49 miRNAs. Among these miRNAs, the targeting effects of PGM1 and miR-30d are the most significant. Importantly, miR-128, miR-130a, miR-150 and miR-873 may play a critical role in this network. The specific mirSVR score can be found in Additional file 3.

## Discussion

As the age increases, the body unavoidably undergoes the physiological aging. The organs or tissues in the body will be affected in different ways, and the microenvironment will also change to varying degrees [15, 22]. In order to better simulate the process of physiological aging, we extracted MSCs from young (1 to 2 months) and old (15 to 18 months) rats, respectively, as the research object to explore the molecular mechanisms underlying aging. There are distinct morphological and molecular biological differences in young and senescent cells, such as increased cell surface area, decreased aspect ratio, and irregular cell bodies, accompanied by cellular metabolic disorders, decreased autophagy, and cell cycle arrest [23–25]. Consistent herewith, MSCs from old rats exhibited senescence-like characteristics in morphology, including irregular shapes, enlarged and flattened cell bodies. Haematopoietic stem cells from old mice also presented increased cell size [15], consistent with our present results. Further, we found that both MSCs from young and old rats have the phenotypic characteristics of mesenchymal progenitor markers, positive for CD44, CD90, and CD105, and negative for CD31 and CD45. These data were congruent with the findings of Zhao [26] and Asumda et al.[27]. However, MSCs from old rats exhibited attenuated osteogenesis, which was in good agreement with our previous studies.

To further confirm the occurrence of senescence in MSCs from old rats, SA-β-gal staining was performed. Our results showed that the SA-β-gal-positive rate in MSCs from old rats was about 3-higher than that from young rats. The “free-radical theory of ageing” was first put forward by Harman in 1956 [28]. Reactive Oxygen Species (ROS), the main product of oxidative stress, is one of the well-established senescence-triggering mechanisms. ROS plays an essential role in several pathways that lead to cellular senescence, such as p16^INK4a^/Rb and p53/p21 pathways[29]. Moreover, monocyte chemoattractant protein-1 (MCP-1) reinforces the senescence of MSCs derived from umbilical cord blood via activation of the ROS-p38-MAPK- p53/p21 signalling pathway [30]. In our current study, MSCs from old rats displayed increased ROS levels and elevated mRNA and protein expression of senescence markers p16^INK4a^ and Rb1. These results were in accordance with our previous findings in aged rats [4, 18, 19, 31]. Given that multiple biological indicators have been to assess senescence, we concluded that MSC senescence occurred, as the individuals physiologically aged.

As we all know, RNA sequence can comprehensively analyze the transcriptome and genome, which not only greatly expands the scale of transcriptome research, but also provides the basis for more widespread genetic analysis and more effective diagnostic treatment. By comparing the RNA sequence information in MSCs obtained from young and old rats, we were capable of describing primary bone marrow-derived MSC-specific signatures as well as the related pathways. There were 868 up-regulated and 2006 down-regulated DEGs (*P* value < 0.05 and fold change > 1.5) in MSCs from old rats in comparison with those from young rats. The majority of top 10 up-regulated genes could arrest cell cycle and decline cell proliferation. In line with this, we found the proliferation index was lower in the old group, and MSCs from old rats were more vulnerable to stagnate in G1 phase in our previous study [4]. And similar findings were obtained in other ageing models [18, 32]. Among the down-regulated genes, the most differentially expressed gene was secreted frizzled-related protein 1 (SFRP1), which could antagonize the Wnt signalling pathway. It is reported that mouse eyes with SFRP1 expression could accelerate the aging process, resembling those found in pseudoexfoliation syndrome, a known age-related disease [33]. The discrepancy might be attributed to model species variation, different cell culture and laboratory conditions. Other down-regulated genes were association with metabolism and inflammation, respectively. In a study performed by Zeliha et al., IL-6 and MT2A carriers may be more likely to obtain longevity compared to non-carriers, which further validates our results [34]. Taken together, we expected that these DEGs may serve as a critical part of database component for future research on cellular senescence.

To better investigate the regulatory effects of DEGs in MSC senescence, GO and KEGG pathway enrichment analysis were conducted. The GO analysis data indicated that the high expression of DEGs might be implicated in developmental process, cell differentiation, growth factor binding and etc. Many studies have further shown that the impaired propensity to proliferate, the decreased differentiation potential and the declined regenerative capacity may be more evident in stem cells such as MSCs [21, 35, 36], muscle stem cells (MuSCs) [37, 38] and HSCs [39, 40] from aged individuals. The down-regulated DEGs chiefly participate in metabolism process. Our latest research shown that the cellular metabolic level of senescent MSCs has changed significantly by the targeted mRNA enrichment analysis of age-related circRNAs [20]. Consistently, quiescent somatic stem cells maintain a low-rate of metabolism to avoid cellular damage from ROS and ensure life-long tissue renewal abilities [41, 42]. Mitochondrial dysfunction is one of the hallmarks of organismal aging, and mitochondrial function is linked to stem cell maintenance and activation [43, 44]. It has been demonstrated that tricarboxylic acid (TCA) cycle, oxidative phosphorylation (OXPHOS) pathways and mitochondrial function were down-regulated pathways in aged MuSCs [45]. Furthermore, maintaining healthy mitochondria by replenishing NAD^+^ stores seemed to protect MuSCs from aging [45]. In accordance to these data, herein, our results showed that up-regulated DEGs were more conducive to cell proliferation and differentiation, while down-regulated genes were mainly related to cell metabolism.

KEGG is a database used to understand the advanced functions and utilities of biological systems from molecular-level information. To better understand the age-related signaling pathways, we conducted KEGG analysis of DEGs. Such analysis comprehensively links DEGs to possibly affected pathways. Consistent with the signaling pathway of up-regulated DEGs enriched, the previous work has found that activating PI3K-AKT pathway through multiple components lead to induction of oncogene-induced senescence (OIS), which may be correlated with the p53/p21^WAF1^ dependent pathway [46, 47]. In line with this, Liu et al. have also shown that activating AKT signaling may delay hair follicle- (HF-) derived-MSC senescence [48]. On the contrary, the down-regulated DEGs were enriched in metabolic pathways, carbon metabolism, glycolysis/gluconeogenesis and PPP. The TCA cycle-associated malic enzyme was capable of suppressing senescence in normal diploid fibroblast IMR90 cells [49]. Glucose 6-phosphate dehydrogenase (G6PD) is the first enzyme and rate-limiting enzyme of the PPP. After oxidant treatment, G6pd-deficient ESCs also enter a state of oxidative stress, which will greatly facilitate the level of cellular ROS and facilitate cell senescence and eventually cause cell death [50]. These evidences are consistent with our analysis results. Collectively, both analyses reveal that genes involved in the metabolism play a critical role in stem cell senescence.

Energy metabolism is traditionally considered as a reactive homeostatic system addressing stage specific cellular energy requirements. Unlike most differentiated mature cells, stem cells mainly produce ATP through glycolysis [5, 51, 52]. In agreement with this, MSC metabolism is also dominated by glycolysis [9, 10]. And enrichment analysis of DEGs displayed that the metabolism in old groups showed a significant reduction compared to that in young groups. Therefore, we speculate that the glycolysis level of senescent MSCs is reduced compared with that of young MSCs. To further verify the decreased glycolytic level of MSCs in old rats, we selected the genes from the glycolysis/gluconeogenesis pathway, which ranked 6th in the KEGG pathway enrichment analysis. The gene selection criteria include relatively high expression level; a cut‐off of absolute *P* value < 0.5 between two groups; KEGG pathway; or published literatures indicate that this gene might be associated with glycolysis processes. The results showed that the expression of catalytic enzymes in the glycolytic pathway such as LDHB, HK1, GPI, PFKM, PFKL, PK, LDHA, ALDOC, PGAM1, HK2, GAPDH, TPI1, ALDOA, and ENO1 were significantly decreased in MSCs from old rats. Of note, 4 of the top 10 genes were key rate-limiting enzymes of glycolysis, including HK1, PFKM, PFKL and PK. To validate the gene expression patterns obtained from RNA-seq, RT-qPCR was performed. The expression of HK1, PFKM, LDHA, LDHB, TPI and PGAM1 are consistent with the sequencing results, while the expression levels of ENO1, ALDOA and PK mRNA were increased. Contrary to the sequencing results, there is no obvious change in HK2 expression. Taken together, these data suggest that glycolysis may be involved in MSC senescence and exerts modulatory functions.

To verify the lower levels of glycolytic metabolism in MSCs from old groups, glucose uptake and lactate levels were firstly examined in both young and senescent MSCs. The results implied that there was markedly decreased glucose consumption by senescent MSCs. Moreover, ATP production and the ECAR level were also substantially diminished in MSCs from old rats. Gitenay and his colleagues also found that senescent human epithelial cells displayed a declined glucose uptake, lactate production as well as lower ATP levels [53]. Inconsistent herewith, it was reported that cell senescence is associated with a progressive and markedly increased rate of glucose metabolism through glycolysis [54, 55]. Metabolite analysis revealed that senescent fibroblasts showed a relative increase in glycolysis [56] and glycolysis-related enzyme activities such as HK, phosphoglycerate kinase (PGK) and PGM were also significantly increased [57]. These data contradict our study. It was well-known that different types of cells have different metabolic patterns and pathways. Since young cells are actively proliferating and senescent cells are growth-arrested, proliferating cells generally exhibit a highly active glycolytic metabolism as compared with arrested cells. Additionally, distinct culture conditions and the number of cell passages in vitro would also lead to this contradictory result. A single cell type and no in vivo experiments are the shortcomings of this study. Further research will be critical for continuing to approach actual clinical practice and exploring the metabolic varieties of aging individuals in vivo. 2-DG, a glucose analogue, inhibits glycolysis via its action on hexokinase, the rate limiting step of glycolysis. After 2-DG treatment, the glycolysis levels were all blunted in MSCs from young and old rats, and the alterations were more conspicuous in MSCs from old rats. In the presence of 2-DG, ESCs also grew more slowly and the ECAR decreased to a greater extent [58], which were consistent with our results. Collectively, these observations suggest that attenuated glycolytic metabolism could contribute to stem cell senescence. Maintenance of glycolysis homeostasis may be a potential target for the preventing and postponing aging.

Many miRNAs play vital roles as regulators in the aging process and are expected to be used as senescence biomarkers [21]. In a study performed by Yang et al., miR-128 directly targeted PFKL in lung cancer cells and influenced the expression of PFKL at both the mRNA and protein levels [59]. Correspondingly, over-expression of miR-128 reduced the glucose uptake and lactate secretion, as well as decreased cellular ATP production [59]. This work provides support for probing the modulatory roles of miR-128- PFKL axis in stem cell senescence. In our previous study, we found that miR-34 expression levels incremented obviously in senescent MSCs, which exerted regulatory functions on both MSC replicative senescence and natural senescence [21]. In good agreement with our previous studies, we herein discovered that miR-34a/c could target PGM1 and further modulate MSC senescence by affecting glycolysis, which is worthy of further exploration.

## Conclusions

In summary, the present study showed the profiles of DEGs in age-associated MSC senescence by RNA sequencing and specific signaling pathways manipulating senescence by function enrichment analysis. Further, our data comprehensively validate that the glycolysis level and the expression of glycolytic enzymes are both decreased in MSCs from old rats. This study naturally opens new avenues for exploring the relationships between stem cell senescence and glucose metabolism. We expand our knowledge of senescence-regulated gene network in rat diploid cells, and uncover a novel role of metabolism in regulating stem cell senescence. The novel factors and pathways identified may be targeted to develop new treatments for aging and age-related disorders. Nonetheless, aging is a multifactorial process, and it is affected not only by several genes but also by the microenvironment and individual lifestyle. The complex metabolic mechanisms regulating MSC senescence and its roles in vivo are worthy of in-depth exploration. Further investigations are necessary to understand the precise mechanisms involved in metabolic regulation on MSC senescence.

## Methods

### Animals and cell cultures

MSCs were isolated from the bone marrows of healthy, male, young (1–2-month-old) and old (15–18-month-old) Wistar rats, obtained from the Experimental Animal Center of Jilin University. The cells were cultured in Dulbecco’s modified Eagle’s medium with nutrient mixture F-12 (DMEM-F12; HyClone, USA) supplemented with 10% fetal bovine serum (FBS; Gibco, USA), 100 U/mL penicillin, and 100 μg/mL streptomycin at 37 °C with 5% CO_2_ in 10 cm cell culture flasks. The medium was replaced every 3 days. Cells were dissociated using 0.25% trypsin (Sigma, USA) and reseeded into multi-well plates for the next stage of the experiments.

### Identification of MSCs surface marker

Briefly, 1 × 10^6^ MSCs were harvested, washed with phosphate-buffered saline (PBS) and incubated with primary antibodies against rat CD44, CD45 (Cell Signal, USA), CD90, CD105 (Millipore, USA), and CD31 (Abcam, UK) overnight at 4 ℃, respectively. Then cells were washed twice with PBS and incubated with secondary antibodies of Fluorescein isothiocyanate (FITC, Proteintech, USA) for 1 h at room temperature (RT), and finally analyzed on a FACSCalibur (BD, USA) for the expression of specific surface markers.

### Osteogenic differentiation assay

MSCs were seeded in 24-well plates at 2.5 × 10^4^ cells per well, as previously described [18, 21]. When the cells reached 70% confluence, the medium was replaced with osteogenic medium. After 3 weeks of induction, the cells were fixed with 4% paraformaldehyde for 30 min, and then stained with Alizarin Red S solution for 5 min at RT to observe bone matrix mineralization. Further, 10% cetylpyridinium chloride (Sigma, USA) was added to quantify the mineralization. The absorbance was measured by a kinetics ELISA reader (Thermo Labsystems, Finland) at 560 nm.

### Senescence-associated β-galactosidase (SA-β-gal) activity assay

To evaluate MSC senescence, SA-β-gal staining was performed using a senescence cell histochemical staining kit (Beyotime, China) on the basis of the manufacturer’s instructions. Briefly, cells were fixed in fixation buffer for 15 min at RT and washed thrice with PBS. After incubation in Staining Solution Mix for 12 h at 37 ℃ without CO_2_, the percentage of β-galactosidase-positive cells (senescent cells) was quantified using a bright-field microscope (OLYMPUS, Japan), assessing randomly at least 200 cells in ten different microscopic fields.

### Measurement of intracellular ROS

The intracellular accumulation of ROS was measured using a Dihydroethidium (DHE) kit (Beyotime, China) on the basis of the manufacturer’s instructions. Briefly, a total of 1 × 10^5^ cells were seeded in 24-well plates and incubated with 10 μM DHE per well for 30 min at 37 °C. Then, remove the medium, wash the cells with serum-free culture medium, and capture the fluorescence images by a fluorescence microscopy (excitation 300 nm; emission 610 nm) (OLYMPUS, Japan). Finally, the images were quantified with Image J software.

### Western blot analysis

Total protein was extracted by protein lysis solution (RIPA: PMSF = 100:1) (Beyotime, China) and concentration was measured using the BCA Protein Assay Kit (Beyotime, China). Then 25 μg of protein were separated by sodium dodecyl sulfate (SDS)-PAGE and transferred onto PVDF membranes (Millipore, USA) by electroblotting. The blotted membranes were blocked for 1 h in 5% non-fat milk at RT and then were probed with anti-p16 ^INK4a^ (1:1,000, Abclonal Techonogy, USA), anti-phospho-Rb1 (1:1,000, Abclonal Techonogy, USA) and anti-β-actin (1:1500 dilution, Abcam, UK) diluted by TBS overnight at 4° C. Membranes were washed three times by TBS and incubated with anti-rabbit IgG secondary antibody y (1:2000 dilution, Proteintech, USA) for 1 h at RT. Electro-Chemi-Luminescence detection system (JENE, UK) was used to detect the protein blots and statistical analysis was performed by Image J software.

### Measurement of glucose uptake and lactate secretion

The cells were seeded in 96-well plates at 3.5 × 10^3^ cells per well. After overnight incubation at 37 ℃, 5% CO_2_, the complete medium was changed to fresh DMEM/F-12 (50 μl/well). After 24 h, the supernatant of cells was collected by centrifugation. Then, according to the manufacturer's instructions, the glucose uptake and lactate production were determined using glucose assay (Jiancheng Bio, Nanjing, China) and lactate assay kits (Jiancheng Bio, Nanjing, China), respectively. Amounts of glucose that remained in the cell culture medium equaled the subtraction of the consumption by cells from total glucose level in an uncultured medium.

### Determination of intracellular ATP production

Intracellular ATP production was measured with the ATP assay kit (Jiancheng Bio, Nanjing, China), as per the manufacturer's protocol. In brief, cells were seeded in the 6-well plate for 12–24 h. Then cells were harvested by using 200–300 μl lysis buffer and vortexed for 1 min. The supernatant was mixed with detection solution and then analysis for ATP concentration was normalized to the corresponding total protein amounts from each sample.

### Extracellular acidification rates (ECAR) analysis

The rates of extracellular acidification were determined using the pH-sensitive (pH-Xtra) (Luxcel Bioscience, Cork, Ireland). Briefly, MSCs obtained from young and old rats were seeded in 96-well plates at 4 × 10^4^ cells/well. After overnight incubation at 37℃, 5% CO_2_, the medium in all the wells was replaced with a preheated liquid mixture containing reconstituted pH-Xtra reagent, fresh culture medium and the different treatments. Then the plate was incubated in a humidified incubator at 37 ℃ for 3–4 h. The fluorescence signal was measured using a CLARIOstar microplate reader (Labtech, Germany).

### Gene expression analysis

Total RNA was extracted from MSCs using RNAiso (Takara, China), and reverse transcription was performed using RNA PCR Kit (AMV) Ver.3.0 (Takara, China). Gene expression levels were determined using real time PCR with TransStart Top Green qPCR SuperMix (TRANS, China) in a 7300 Real-Time PCR System (ABI, USA). β-actin served as an internal standard. The primer sequences were listed in Table 4, and relative transcript levels were calculated using the 2^−ΔΔCt^ method.

**Table 4:** Primers used for Real-time RT-PCR

Gene	Forward primer sequence	Reverse primer sequence
β-actin	GGAGATTACTGCCCTGGCTCCTA	GACTCATCGTACTCCTGCTTGCTG
P16^INK4A^	AACACTTTCGGTCGTACCC	GTCCTCGCAGTTCGAATC
Rb1	CATGCTAGCAGCTGTTCTTTAC	AGTTTGCCTATATCAGCACAGT
ALDOA	TGTTGTGGGCATTAAGGTAGA	GAGGGACGAGGGAGTATGC
ENO1	CAAGTCCTTCATCAAGAACTATCC	TGTCAGGTCATCTCCCACAAT
HK1	TCTAAACTCTGGGAAACAAAGGT	GAAGTCAATCAGGATGTTACGG
HK2	GCCGTAGTGGACAAGATAAGAGAG	GCCTCCATACCCATCTTGCTAC
LDHA	TCCCTGAAGTCTCTGAACCCG	TCCTCCTTGATTCCATAGAGACC
LDHB	GGTGGTGGACAGTGCCTATGA	GACTTCGTTCTCGATGCCGTA
PFKM	GTGAAAGACCTGGTGGTTCAGA	CTCTGAGATCTTGCCTTCCTTC
PK	GAAACAGCCAAAGGGGACTA	GGACTCCGTCAGAACTATCAAA
PGAM1	GAGCGACACTATGGGGGTCTAAC	TCTGCGTACCTGCGGTCCTT
TPI1	CACTCTGAACGCAGCCAAGC	CACCCAGGTAGCTCCTAAGTCC

### RNA sequence library construction

Agarose gel electrophoresis was used for the integrity and quality testing of total RNA. Then, 1–2 μg total RNA was used to construct sequencing libraries by KAPA Stranded RNA-Seq Library Prep Kit (Illumina, America) according to the manufacturer’s instructions. The constructed library was quality tested by Agilent 2100 Bioanalyzer (Agilent Technologies, America), and the library quantified using qPCR.

### RNA sequencing

The mixed sample sequencing library was transformed by NaOH to generate single-stranded DNA and diluted to a concentration of 8 pM. Then amplified by the TruSeq SR Cluster Kit (Illumina, America), and the ends of the generated fragments were sequenced 150 cycles through Illumina HiSeq 4000 (Illumina, America).

### RNA sequencing data processing

Sequencing quality was assessed by FastQC software and bases were compared to the genome by Hisat2 software. Then, referring to the official database, bases were annotated with information via StringTie software and transcript abundance was estimated. Fragments per kilobase of transcript per million mapped reads (FPKM) values for gene expression levels were calculated and differentially expressed genes (DEGs) were identified by R programming language. Finally, principal component analysis (PCA), Hierarchical Clustering, Gene Ontology, Pathway analysis, scatter plots and volcano plots were performed with the DEGs for statistical computation and graphics.

### Functional enrichment analysis

Gene Ontology (GO) is an international standard classification system for gene function. Based on discrete distribution, significant analysis false positive rate analysis, and enrichment analysis of classification results were carried out, and gene function classification with significant correlation and targeting were obtained. Pathway analysis is a method of studying the interactions between multiple proteins to collectively regulate cellular functions and metabolic processes. According to the latest Kyoto Encyclopedia of Genes and Genomes (KEGG) database, biological pathways closely related to differentially expressed genes were identified and significant analysis were performed. Significant GO terms and KEGG pathways were selected according to FDR corrected *P* values < 0.05.

### MiRNA prediction of glycolytic related genes

Online database (microRNA.org) for miRNA prediction was applied to predict the potential miRNAs of glycolytic related genes. PhastCons indicates the evolutionary conservation of gene untranslated regions in various species. And mirSVR stands for thermodynamic stability, indicating the stability of the combination of miRNA-mRNA. Target miRNAs were selected according to PhastCons ≥ 0 and mirSVR Score ≤ − 0.1. The information of mRNAs, miRNAs and mirSVR score were imported and the networks were illustrated using Cytoscape software version 3.7.1.

### Statistical analysis

Data were represented as means ± SD of three independent experiments. Two groups were compared by a two-tailed Student’s t-test. Differences were considered to be statistically significant at *P* < 0.05. *P < 0.05, **P < 0.01, ***P < 0.001.

## Supplementary Information

**Additional file 1: **The top 5 up/downregulated GO terms.

**Additional file 2: **The top 10 up/downregulated Pathways.

**Additional file 3: **The details of glycolysis related miRNAs.

**Additional file 4: Figure S1.** Characteristics of MSCs from young and old rats. (A) The morphology of MSCs at passage 1 (P1) and passage 3 (P3) from young rats. Scale bar, 100 µm. (B) SA-β-gal staining and quantification of β-gal positive cell numbers for P1 and P3 MSCs from young rats. Scale bar, 50 μm. (C) The phenotypic detection of MSCs from young and old rats by flow cytometry. All data were presented as mean ± SEM (error bars), n = 3, N.S. indicated no statistical value.

## Data Availability

Not applicable.

## Abbreviations

2-DG: 2-Deoxy-D-glucose
AKR1C14: Aldo–keto reductase family 1, member C14
ALDOA: Aldolase-fructose-bisphosphate
ALDOC: Aldolase-fructose-bisphosphate C
CCL20: C–C motif chemokine ligand 20
CILP: Cartilage intermediate layer protein
CircRNAs: Circular RNAs
DEGs: Differentially expressed genes
DHE: Dihydroethidium
ECAR: Relative extracellular acidification rates
ENO1: Enolase 1
FHL1: Four and a half LIM domains 1
FITC: Fluorescein isothiocyanate
FPKM: Fragments per kilobase of transcript per million mapped reads
G6PD: Glucose 6-phosphate dehydrogenase
GAPDH: Glyceraldehyde-3- phosphate dehydrogenase
GO: Gene Ontology
GPI: Glucose-6-phosphate isomerase
HK1: Hexokinase 1
HK2: Hexokinase 2
HSCs: Hematopoietic stem cells
IL1RN: Interleukin 1 receptor antagonist
KEGG: Kyoto Encyclopedia of Genes and Genomes
LDHA: Lactate dehydrogenase A
LDHB: Lactate dehydrogenase B
MCP-1: Monocyte chemoattractant protein-1
MiRNAs: MicroRNAs
MSCs: Mesenchymal stem cells
MT2A: Metallothionein 2A
MuSC: Muscle stem cells
MYH11: Myosin heavy chain 11
NAD^+^: Intracellular nicotinamide adenine dinucleotide
NAFLD: Non-alcoholic fatty liver disease
OIS: Oncogene-induced senescence
OXPHOS: Oxidative phosphorylation
PBS: Phosphate-buffered saline
PCA: Principal component analysis
PFKL: Phosphofructokinase in liver
PFKM: Phosphofructokinase in muscle
PGAM1: Phosphoglycerate mutase 1
PK: Pyruvate kinase
PPP: Pentose phosphate pathway
RBP4: Retinol binding protein 4
RNA-seq: RNA sequencing
ROS: Reactive oxygen species
SA-β-gal: Senescence-associated β-galactosidase
SDS: Sodium dodecyl sulfate
SFRP1: Secreted frizzled-related protein 1
SLC1A3: Solute carrier family 1 member 3
TCA: Tricarboxylic acid
TGFB3: Transforming growth factor, beta 3
TPI: Glyceraldehyde-3-phosphate dehydrogenase
WHO: World Health Organization
