# Supplementary material for: RNA sequencing profiles reveal dynamic signaling and glucose metabolic features during bone marrow mesenchymal stem cell senescence
Source: Cell Biosci. 2022 May 14;12:62. doi: 10.1186/s13578-022-00796-5 (PMC9107734; doi:10.1186/s13578-022-00796-5)
Supplement: Supplementary file 1 — Additional file 1: The top 5 up/downregulated GO terms. [file 13578_2022_796_MOESM1_ESM.pdf]

Additional File 1. The top 5 up/downregulated GO terms.

	ID	Term	*P* value	FDR	ES	FE
**Upregulated**						
Biological Process	GO:0009653	Anatomical structure morphogenesis	4.194E-40	2.210E-36	39.377	2.474
	GO:0007275	Multicellular organism development	5.975E-35	1.574E-31	34.224	1.764
	GO:0048856	Anatomical structure development	3.874E-34	6.805E-31	33.412	1.710
	GO:0009888	Tissue development	7.696E-34	1.014E-30	33.114	2.563
	GO:0048731	System development	1.468E-33	1.547E-30	32.833	1.801
Cellular Component	GO:0005912	Adherens junction	1.241E-26	7.771E-24	25.906	4.894
	GO:0030055	Cell-substrate junction	2.868E-26	8.978E-24	25.542	6.770
	GO:0031012	Extracellular matrix	2.816E-24	5.386E-22	23.550	4.524
	GO:0005924	Cell-substrate adherens junction	3.442E-24	5.386E-22	23.463	6.606
	GO:0005925	Focal adhesion	5.720E-23	6.878E-21	22.243	6.544
Molecular Function	GO:0005515	Protein binding	3.449E-24	3.584E-21	23.462	1.432
	GO:0042277	Peptide binding	1.417E-23	7.364E-21	22.848	1.419
	GO:0033218	Amide binding	2.349E-23	8.136E-21	22.629	1.415
	GO:0008092	Cytoskeletal protein binding	8.186E-19	1.824E-16	18.087	2.605
	GO:0003779	Actin binding	8.777E-19	1.824E-16	18.057	3.714
**Downregulated**						
Biological Process	GO:0009058	Biosynthetic process	2.176E-28	1.572E-24	27.662	1.571
	GO:0044237	Cellular metabolic process	5.532E-25	1.998E-21	24.257	1.269
	GO:0051186	Cofactor metabolic process	6.753E-23	1.626E-19	22.171	2.667
	GO:0044238	Primary metabolic process	6.073E-21	1.097E-17	20.217	1.257
	GO:0006412	Translation	8.308E-21	1.200E-17	20.080	2.510
Cellular	GO:0005737	Cytoplasm	9.928E-35	8.766E-32	34.003	1.246

Component	GO:0005622	Intracellular	1.811E-32	7.996E-30	31.742	1.158
	GO:0005840	Ribosome	2.741E-24	8.067E-22	23.562	2.783
	GO:0044391	Ribosomal subunit	5.494E-21	1.213E-18	20.260	2.802
	GO:0043229	Intracellular organelle	1.263E-19	2.231E-17	18.899	1.161
Molecular Function	GO:0003735	Structural constituent of ribosome	3.484E-27	6.378E-24	26.458	3.199
	GO:0003824	Catalytic activity	8.394E-16	7.684E-13	15.076	1.265
	GO:0016491	Oxidoreductase activity	1.475E-14	9.004E-12	13.831	1.911
	GO:0005198	Structural molecule activity	4.651E-11	2.129E-08	10.332	1.751
	GO:0050662	Coenzyme binding	1.360E-10	4.982E-08	9.866	2.293

Abbreviation: ES, Enrichment Score value of the term, it equals"-log10(*P* value)"; FDR, the false discovery rate of the term, using Benjamini & Hochberg (1995) method; FE, Fold Enrichment value of the term, it equals (Count/Size)/(numInt/numTotal), ("numInt" stands for the total number of DE genes; "numTotal" stands for the total number of background population genes); *P* value stands for the fisher exact test value of the term.
