# Supplementary material for: RNA sequencing profiles reveal dynamic signaling and glucose metabolic features during bone marrow mesenchymal stem cell senescence
Source: Cell Biosci. 2022 May 14;12:62. doi: 10.1186/s13578-022-00796-5 (PMC9107734; doi:10.1186/s13578-022-00796-5)
Supplement: Supplementary file 2 — Additional file 2: The top 10 up/downregulated Pathways. [file 13578_2022_796_MOESM2_ESM.pdf]

Additional File 2. The top 10 up/downregulated Pathways.

ID	Term	*P* value	FDR	ES
**Upregulated**				
rno04510	Focal adhesion	3.120E-21	7.425E-19	20.506
rno04512	ECM-receptor interaction	1.093E-14	1.301E-12	13.961
rno05205	Proteoglycans in cancer	1.126E-09	8.937E-08	8.948
rno04810	Regulation of actin cytoskeleton	1.326E-08	7.892E-07	7.877
rno04151	PI3K-Akt signaling pathway	3.578E-07	1.598E-05	6.446
rno05146	Amoebiasis	4.028E-07	1.598E-05	6.395
rno05414	Dilated cardiomyopathy	7.018E-07	2.134E-05	6.154
rno04390	Hippo signaling pathway	7.173E-07	2.134E-05	6.144
rno05200	Pathways in cancer	1.643E-06	4.346E-05	5.784
rno05410	Hypertrophic cardiomyopathy (HCM)	1.908E-06	4.540E-05	5.720
**Downregulated**				
rno03010	Ribosome	1.906E-28	5.871E-26	27.720
rno01100	Metabolic pathways	8.016E-15	1.235E-12	14.096
rno01200	Carbon metabolism	1.824E-07	1.873E-05	6.739
rno04932	Non-alcoholic fatty liver disease (NAFLD)	3.295E-06	2.537E-04	5.482
rno00051	Fructose and mannose metabolism	5.246E-06	3.231E-04	5.280
rno00010	Glycolysis/Gluconeogenesis	6.416E-06	3.294E-04	5.193
rno00030	Pentose phosphate pathway	2.354E-05	1.036E-03	4.628
rno04146	Peroxisome	4.426E-05	1.704E-03	4.354
rno04066	HIF-1a signaling pathway	4.997E-05	1.710E-03	4.301
rno01230	Biosynthesis of amino acids	7.069E-05	2.177E-03	4.151

Abbreviation: ES, Enrichment Score value of the pathway, it equals "-log10(*P* value)"; FDR, false discovery rate of the pathway; *P* value stands for the enrichment *P* value of the pathway used Fisher exact test.
