# Supplementary material for: RNA sequencing profiles reveal dynamic signaling and glucose metabolic features during bone marrow mesenchymal stem cell senescence
Source: Cell Biosci. 2022 May 14;12:62. doi: 10.1186/s13578-022-00796-5 (PMC9107734; doi:10.1186/s13578-022-00796-5)
Supplement: Supplementary file 3 — Additional file 3: The details of glycolysis related miRNAs. [file 13578_2022_796_MOESM3_ESM.pdf]

Additional File 3. The details of glycolysis related miRNAs.

mRNA	miRNA	mirSVR
LDHB	mir-23a	-0.2246
	mir-23b	-0.4297
	mir-215	-0.2949
	mir-192	-0.2949
	mir-301a	-0.8687
	mir-301b	-0.8687
	mir-130a	-0.885
	mir-130b	-0.885
	mir-19a	-0.4297
	mir-19b	-0.4052
	mir-28	-0.2028
	mir-708	-0.2066
PGM1	mir-455	-0.17
	mir-34a	-0.2388
	mir-34c	-0.241
	mir-449a	-0.2431
	mir-150	-0.2709
	mir-31	-0.4023
	mir-342	-0.3471
	mir-142	-0.9864
	mir-27a	-0.7392
	mir-27b	-0.7392
	mir-873	-0.7487
	mir-128	-1.0491
	mir-384	-1.1717
	mir-30a	-1.1889
	mir-30b	-1.1889
	mir-30c	-1.1889
	mir-30d	-1.1906
	mir-30e	-1.1872
	mir-124	0.4796
	mir-143	-0.6619
	mir-539	-0.9551
GPI	mir-152	-0.2342
	mir-148	-0.2342
	mir-130	-0.1495
	mir-150	-0.7153
	mir-330	-0.4428
	mir-326	-0.4428
	mir-485	-0.2541
PFKM	mir-383	-0.6889
	mir-200a	-0.591

	mir-141	-0.5947
	mir-128	-0.1093
	mir-542	-0.9581
	mir-320	-0.3941
	mir-186	-0.1457
PFKL	mir-873	-0.1866
	mir-346	-0.1907
	mir-125a	-0.8547
	mir-1	-0.2566
	mir-206	-0.2566
