# Supplementary figures and images for: RNA sequencing profiles reveal dynamic signaling and glucose metabolic features during bone marrow mesenchymal stem cell senescence
Source: Cell Biosci. 2022 May 14;12:62. doi: 10.1186/s13578-022-00796-5 (PMC9107734; doi:10.1186/s13578-022-00796-5)

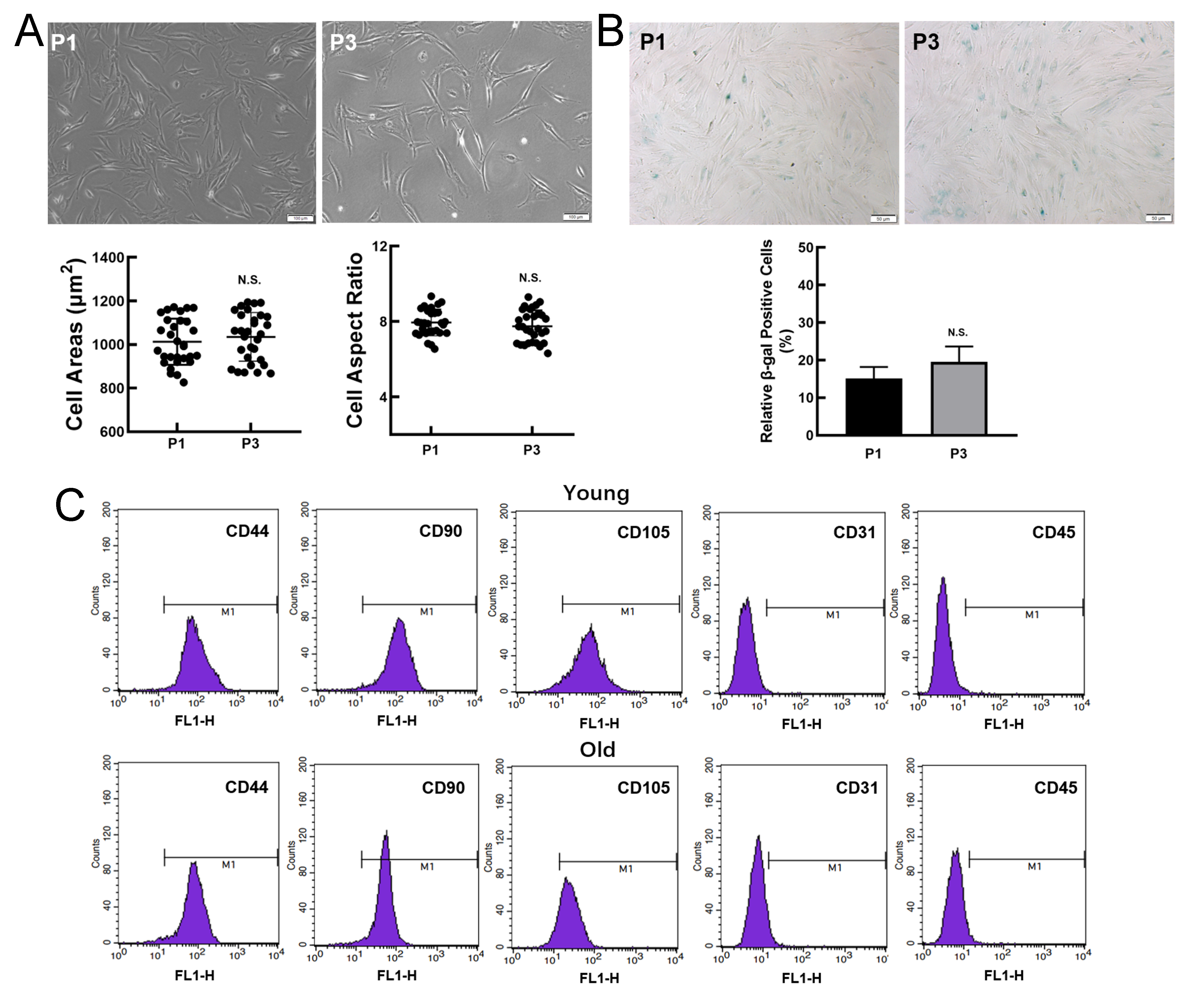

Supplement: Supplementary file 4 — Additional file 4: Figure S1. Characteristics of MSCs from young and old rats. (A) The morphology of MSCs at passage 1 (P1) and passage 3 (P3) from young rats. Scale bar, 100 µm. (B) SA-β-gal staining and quantification of β-gal positive cell numbers for P1 and P3 MSCs from young rats. Scale bar, 50 μm. (C) The phenotypic detection of MSCs from young and old rats by flow cytometry. All data were presented as mean ± SEM (error bars), n = 3, N.S. indicated no statistical value. [file 13578_2022_796_MOESM4_ESM.tif]
